# One-Month Diesel Exhaust Inhalation Produces Hypertensive Gene Expression Pattern in Healthy Rats

**DOI:** 10.1289/ehp.11647

**Published:** 2008-09-12

**Authors:** Reddy R. Gottipolu, J. Grace Wallenborn, Edward D. Karoly, Mette C. Schladweiler, Allen D. Ledbetter, Todd Krantz, William P. Linak, Abraham Nyska, Jo Anne Johnson, Ronald Thomas, Judy E. Richards, Richard H. Jaskot, Urmila P. Kodavanti

**Affiliations:** 1 Experimental Toxicology Division, National Health & Environmental Effects Research Laboratory, Office of Research and Development, U.S. Environmental Protection Agency, Research Triangle Park, North Carolina, USA; 2 School of Public Health, University of North Carolina, Chapel Hill, North Carolina, USA; 3 Human Studies Division, National Health & Environmental Effects Research Laboratory, Office of Research and Development, U.S. Environmental Protection Agency, Chapel Hill, North Carolina, USA; 4 Air Pollution Prevention and Control Division, National Risk Management Research Laboratory, Office of Research and Development, U.S. Environmental Protection Agency, Research Triangle Park, North Carolina, USA; 5 Tel Aviv University, Tel Aviv, Israel; 6 Laboratory of Experimental Pathology, National Institute of Environmental Health Sciences, National Institutes of Health, Department of Health and Human Services, Research Triangle Park, North Carolina, USA

**Keywords:** air pollution, cardiac gene expression profile, diesel exhaust, hypertension, mitochondria, particulate matter

## Abstract

**Background:**

Exposure to diesel exhaust (DE) is linked to vasoconstriction, endothelial dysfunction, and myocardial ischemia in compromised individuals.

**Objective:**

We hypothesized that DE inhalation would cause greater inflammation, hematologic alterations, and cardiac molecular impairment in spontaneously hypertensive (SH) rats than in healthy Wistar Kyoto (WKY) rats.

**Methods and results:**

Male rats (12–14 weeks of age) were exposed to air or DE from a 30-kW Deutz engine at 500 or 2,000 μg/m^3^, 4 hr/day, 5 days/week for 4 weeks. Neutrophilic influx was noted in the lung lavage fluid of both strains, but injury markers were minimally changed. Particle-laden macrophages were apparent histologically in DE-exposed rats. Lower baseline cardiac anti-oxidant enzyme activities were present in SH than in WKY rats; however, no DE effects were noted. Cardiac mitochondrial aconitase activity decreased after DE exposure in both strains. Electron microscopy indicated abnormalities in cardiac mitochondria of control SH but no DE effects. Gene expression profiling demonstrated alterations in 377 genes by DE in WKY but none in SH rats. The direction of DE-induced changes in WKY mimicked expression pattern of control SH rats without DE. Most genes affected by DE were down-regulated in WKY. The same genes were down-regulated in SH without DE producing a hypertensive-like expression pattern. The down-regulated genes included those that regulate compensatory response, matrix metabolism, mitochondrial function, and oxidative stress response. No up-regulation of inflammatory genes was noted.

**Conclusions:**

We provide the evidence that DE inhalation produces a hypertensive-like cardiac gene expression pattern associated with mitochondrial oxidative stress in healthy rats.

Traffic-related ambient particulate matter (PM) exposures, including diesel exhaust (DE), are relevant because of increased risk of cardiovascular mortality in populations residing close to highways with dense vehicular transit (Tonne et al. 2007). Cardiac ischemia and myocardial infarction have been associated with DE and traffic-related PM exposures in healthy and compromised humans (Mills et al. 2007; Tonne et al. 2007). A number of studies have also demonstrated vasoconstriction, endothelial dysfunction, thrombosis, and hypertension in humans and disease-prone animals (Brook 2005, 2007; Campen et al. 2005; Carlsten et al. 2007; Hansen et al. 2007; Mills et al. 2005, 2007; Peretz et al. 2008; Tornqvist et al. 2007; Urch et al. 2005). Most of these effects have been observed after acute exposure. It is not known if cardiac effects of DE are independent of or secondary to pulmonary inflammation and vascular activation/thrombosis.

The prevalent mechanistic explanations of how DE exposure causes health effects include involvement of oxidative stress. DE contains a variety of redox-cycling quinones and poly-cyclic aromatic hydrocarbons (Shimada et al. 2004). A number of *in vitro* and *in vivo* studies have demonstrated that DE produces oxidative stress and inflammation in the lung and systemic vasculature (Sagai et al. 1993). Although reactive organic DE constituents likely translocate to the systemic circulation (Persson et al. 2002), it is not clear if extra-pulmonary effects are mediated by interaction of DE with vasculature.

Oxidative stress has been demonstrated as a major risk factor for chronic diseases, including cardiovascular disease (Abrescia and Golino 2005). To understand the role of underlying oxidative stress in exacerbation of PM cardiopulmonary toxicity, we have used the spontaneously hypertensive (SH) rats in several of our studies (Kodavanti et al. 2000; 2002; Wallenborn et al. 2007). We have shown that pulmonary oxidative stress, vascular leakage, and systemic effects of PM are exacerbated in SH relative to normotensive parental Wistar Kyoto (WKY) rats. However, we could not demonstrate greater incidence of cardiac pathology after subchronic exposure to combustion source PM (Kodavanti et al. 2003). SH rats demonstrate a host of abnormalities, including increased systemic oxidative stress and mitochondrial impairment in the heart (Zhou et al. 2008). In this study, we hoped to gain insight into how cardiovascular susceptibilities of SH and WKY rats differ after DE exposure.

Antioxidant enzymes play a crucial role in compensatory response after tissue injury. These enzymes themselves are vulnerable to inactivation by increased free radicals resulting in inhibition (Tsutsui et al. 2006; Ullrich and Kissner 2006). The enzymes of the tricarboxylic acid cycle and electron transport chain within mitochondria are also sensitive to inhibition by reactive oxygen species (Abrescia and Golino 2005; Ullrich and Kissner 2006). Because mitochondria produce the most free radicals during aerobic respiration, especially in the heart, inhibition of activities of iron sulfur cluster-containing mitochondrial enzymes has been used to demonstrate increased oxidative stress (Tsutsui et al. 2006).

Because DE produces oxidative stress (Sagai et al. 1993) and SH rats demonstrate mitochondrial defects with oxidative stress (Leary et al. 2002; Zhou et al. 2008), we hypothesized that, in SH, DE exposure will lead to greater cardiac toxicity and mitochondrial impairment than in WKY rats. We further postulated that cardiac gene expression will be differentially affected by DE in these rats and will provide mechanistic insight into how DE may produce functional impact differently in healthy and hypertensive rats. We exposed WKY and SH rats to DE for 4 weeks and determined biochemical and molecular measures of cardiopulmonary toxicity. We also analyzed cardiac ultrastructural changes, oxidative stress, and gene expression pattern using Affymetrix arrays (Affymetrix Inc., Santa Clara, CA, USA). Our data demonstrate cardiac mitochondrial oxidative stress and development of a hypertensive gene expression pattern in WKY rats, characterized by a generalized suppression of genes that are already suppressed in SH rats at baseline without DE.

## Materials and Methods

### Animals

Healthy male WKY rats, and SH rats, 12–14 weeks of age, were purchased from Charles River Laboratories Inc. (Raleigh, NC, USA). Rats were housed in an animal facility approved by the Association for Assessment and Accreditation of Laboratory Animal Care. Prior to the experimental period, rats were acclimatized for at least 2 weeks under controlled conditions (21 ± 1°C, 50 ± 5% relative humidity, 12-hr light-dark cycle). Use of animals in this study was preapproved by the National Health & Environmental Effects Research Laboratory (NHEERL), U.S. Environmental Protection Agency (EPA) Animal Care and Use Committee. Animals were single housed in polycarbonate individually ventilated cages with beta chip bedding during the experimental period. All animals received standard Purina rat chow (Purina Mills, Brentwood, MO, USA) and water *ad libitum*. Animals were treated humanely and with regard for alleviation of suffering.

### DE generation and exposure characterization

DE was generated by operating a 30-kW (40 hp) 4-cylinder indirect injection Deutz diesel engine (BF4M1008; DEUTZ AG, Koln-Parz, Germany) essentially as described by Stevens et al. (2008) [see Supplemental Material (http://www.ehponline.org/members/2008/11647/suppl.pdf)]. A small portion of the exhaust was routed to a dilution system and passed through two-stage air dilution and was then routed to Hinner exposure chambers. Three Hinner exposure chambers were used (0, 500, and 2,000 μg/m^3^) in parallel. Two chambers pulled directly from the dilution system and contained diluted flue gas and target PM concentrations of 2,000 μg/m^3^, with additional dilution at the second chamber of 500 μg/m^3^. The third chamber (air control) pulled clean, filtered room air [see Supplemental Material (http://www.ehponline.org/members/2008/11647/suppl.pdf)].

In addition to nitric oxide (NO) and DE mass measurements, we used continuous emission monitors (CEMs) to measure chamber concentrations of oxygen (O_2_; model 755, Beckman Corp., La Habra, CA, USA), carbon monoxide (CO; model 48, Thermo Electron Corp., Franklin, MA, USA), nitrogen dioxide (NO_2_; model 42c, Thermo Electron Corp., Franklin, MA, USA), and sulfur dioxide (SO_2_; model 43c; Thermo Electron Corp.). The concentrations of gaseous components varied between two chambers in line with the high and low DE mass concentrations (Table 1). Particle size distributions were characterized using a scanning mobility particle sizer (SMPS; model 3080/3022a; TSI Inc., St. Paul, MN, USA) and an aerodynamic particle sizer (APS; model 3321; TSI Inc.). Chamber temperature and relative humidity were monitored continuously. Integrated 4-hr filter samples (14.1 L/min) were collected daily from each chamber and analyzed gravimetrically to determine particle concentrations. In addition, triplicate quartz filter samples were collected daily from the exposure chambers and analyzed using a thermal/optical carbon analyzer (model 107, Sunset Laboratory Inc., Tigard, OR, USA) to determine organic carbon/elemental carbon (OC/EC) partitioning of the collected particles. A summary of these data are depicted in Table 1. To accommodate all types of tissue analysis using three groups of rats [*n* = 6 for pathology and molecular analysis; *n* = 6 for cardiac mitochondria isolation, and *n* = 3 for cardiac transmission electron microscopy (TEM)], the DE exposures were conducted over 6 weeks (Monday through Friday); each group of animals began on a different day and received a total of 4 weeks of exposure. Thus, the data in Table 1 represent average values of a 6-week (5 days/week) exposure period.

### Exposure of rats to air or DE

Animals were periodically monitored for changes in breathing parameters using barometric whole-body plathysmography system (Buxco Electronics, Inc., Sharon, CT, USA) to obtain data on pulmonary ventilation as described previously (Kodavanti et al. 2005). Each group of rats was exposed to air or DE (500 and 2,000 μg/m^3^), 4 hr/day, 5 days per week for 4 consecutive weeks, and necropsies were performed 1 day after the final exposure.

### Necropsy and sample collection

At designated time points, rats were weighed and anesthetized with intraperitoneal sodium pentobarbital (50–100 mg/kg). Blood was collected from the abdominal aorta directly into blood collection tubes containing EDTA (for complete blood counts), citrate (for plasma protein analysis), or in serum separator tubes without an anticoagulant for cytokine assays. In the first set of animals (*n* = 6), the heart was removed, blotted dry, weighed, and cut into two mid-longitudinal halves. One half was fixed in 10% neutral formalin for histologic evaluation. From the second half, the right ventricle was discarded, and portions of the left ventricle plus septum were snap-frozen in liquid nitrogen and retained for enzyme activity analysis and RNA isolation.

The trachea was cannulated, and the left lung was tied. The right lung was lavaged with Ca^++^/Mg^++^–free phosphate-buffered saline (pH 7.4) as described previously (Wallenborn et al. 2007). The left lung was tracheally fixed with neutral formalin for later histologic evaluation.

### Bronchoalveolar lavage fluid (BALF) analysis

Aliquots of BALF were used to determine total cell counts with a Z1 Coulter Counter (Coulter, Inc., Miami, FL, USA). A second aliquot was centrifuged (Shandon 3 Cytospin, Shandon, Pittsburg, PA, USA) to prepare cell differential slides. Slides were dried at room temperature and stained with Leukostat (Fisher Scientific Co., Pittsburg, PA, USA). Macrophages and neutrophils were counted using light microscopy. At least 300 cells were counted on each slide. The remaining BALF was centrifuged at 1,500 × *g* to remove cells, and the supernatant fluid was analyzed for markers of lung injury. Total protein, albumin, lactate dehydrogenase activity, *N*-acetyl glucosaminidase activity, and γ-glutamyl transferase (GGT) activity were measured as described previously (Wallenborn et al. 2007).

### Blood chemistry and cytology

Aliquots of EDTA-collected blood were analyzed for complete blood counts by a Beckman-Coulter AcT blood analyzer (Beckman-Coulter Inc., Fullerton, CA, USA). Each blood sample containing citrate anticoagulant was centrifuged at 4,500 rpm for 10 min at 4°C. Plasma fibrinogen, activated plasma thromboplastin time, and plasma thromboplastin time were measured in citrated plasma by Laboratory Corporation Inc., Durham, NC, USA. Angiotensin-converting enzyme (ACE) activity was measured using reagents and controls from Sigma Aldrich, St. Louis, MO, USA. C-Reactive protein (CRP) was measured using an SPQ II kit that contained its own calibrations and controls (Diasorin Inc., Stillwater, MN, USA). D-Dimer measurements were performed using a kit obtained from Kamiya Biomedical Company (Seattle, WA, USA). Total antioxidant status was determined using a kit from RANDOX Laboratories Ltd. (Oceanside, CA, USA). These assays were modified and adapted for use on the KONLAB clinical chemistry analyzer (Thermo Clinical Labsystems, Espoo, Finland).

### Determination of cytokines in BALF and serum

Serum and BALF samples were analyzed using a 24 rat cytokine/chemokine Lincoplex Kit (Linco Research Inc., St. Charles, MO, USA). Samples were processed according to protocol using Luminex 100 system (Luminex Corporation, Austin, TX, USA). Sample values were normalized based on standard curve for each protein and data were calculated using Luminex software. Note that not all markers provided positive values for samples analyzed.

### Lung and heart light microscopy and cardiac TEM

Tissues from heart and lung were processed, embedded in paraffin, sectioned at 5 μm, and stained with hematoxylin and eosin (H&E) for pathological analysis as previously described (Nyska et al. 2005). A separate group of rats (*n* = 3 for each control and 2,000 μg/m^3^ group) were anesthetized and exsanguinated via the abdominal aorta. Hearts were quickly removed and processed for TEM [see Supplemental Material (http://www.ehponline.org/members/2008/11647/suppl.pdf)].

### Preparation of cardiac cytosol, mitochondria, and whole homogenates

Cardiac tissues from a separate group of rats (*n* = 6) were excised quickly. The lung tissue was processed as indicated above. The right ventricle was discarded. A small portion of the left ventricle was quick-frozen for later homogenization, and the remaining large portion was homogenized and processed as indicated earlier for isolation of mitochondria and cytosol (Wallenborn et al. 2008).

### Analysis of oxidative stress-sensitive enzyme markers

Activities of aconitase, superoxide dismutase, glutathione peroxidase, glutathione transferase, thio redoxin reductase, isocitrate dehydrogenase, and ubiquinone reductase were measured spectrophotometrically in cytosolic and/or mitochondrial fractions [see Supplemental Material (http://www.ehponline.org/members/2008/11647/suppl.pdf)].

### RNA isolation

Heart total (left ventricle) RNA was isolated from tissues snap-frozen in liquid nitrogen using TriReagent (Sigma). RNA was further purified with Qiagen Rneasy minicolumns (Qiagen, Valencia, CA, USA) and resuspended in 50 μL diethylpyrocarbonate-treated water according to the manufacturer’s protocol. RNA quality was assessed with an Agilent 2100 Bioanalyzer (Agilent Technologies, Palo Alto, CA, USA). All samples had a 28S/18S ratio ≥ 2.0 and were stored at −80°C for gene chip analysis or their use in real-time polymerase chain reaction (PCR).

### Microarray target preparation and hybridization

Expression Analysis Inc. (Durham, NC, USA) performed RNA target preparation and hybridization to the Affymetrix GeneChip Rat 230A microarray containing 15,923 probe sets and expressed sequence tags (Affymetrix Inc.). The process for cRNA synthesis, hybridization, visualization, and quantification are described elsewhere (Gilmour et al. 2006). Fluorescent images were detected in a GeneChip Scanner 3000 (Affymetrix), and expression data were extracted using the default setting in the Microarray Suite 5.0 software (Affymetrix). For microarray purposes, four biological replicates were collected for each group (air and 2,000 μg/m^3^ DE-exposed rats of each strain).

### Real-time PCR

To confirm the Affymetrix gene array data, we performed real-time quantitative PCR for β-actin, hemeoxygenase-1, and endothelin-1 using heart RNA derived from air and DE-exposed WKY and SH rats as described previously (Gilmour et al. 2006). Fold values for expression changes observed using PCR are in general agreement with the values obtained from the microarray data [see Supplemental Material, Table 1 (http://www.ehponline.org/members/2008/11647/suppl.pdf)].

### Statistical analysis

All data other than Affymetrix were analyzed by two-way analysis of variance (ANOVA) with strain and exposure as two factors using SigmaStat software, version 3.5 (Systat Software, Inc., Point Richmond, CA, USA). In the case of significant interaction (*p* < 0.05), stepdown ANOVA was used to test for main effect with DE. Pairwise comparisons between groups were made using the Holm-Sidak method. Statistical significance was stated when a minimum *p*-value of 0.05 or less was reached.

### Statistical analysis for gene chip data

We imported Affymetrix CEL data files into R, an open-source statistical scripting language (R Foundation for Statistical Computing 2008) used in conjunction with the Bioconductor project (Gentleman et al. 2004). Normalized values with Robust Multichip Average background correction, quartile normalization, and median polish were calculated with the R/bioconductor package AffylmGUI (Irizarry et al. 2003). A linear model was fitted to the data and used to average data between replicate arrays and to identify variability between them. Contrasts between groups were used to generate *p*-values, moderated *t* statistics, Empirical Bayes statistics, and M value [log_2_ (ratio)]. Probe sets with a *p*-value < 0.01 after an adjustment with a Benjamini and Hochberg false discovery rate (FDR, 5%) test were judged by the limma package to be significant within group contrasts. The following contrasts were made for this study: SH-air/WKY-air, SH-DE/SH-air, SH-DE/WKY-air, WKY-DE/WKY-air. Probe sets lacking an entrez gene ID or identified as hypothetical proteins were removed from the gene lists.

The microarray data were deposited in the Gene Expression Omnibus Web site (accession number GSE9694; National Center for Biotechnology Information 2008). The heat maps for the differentially expressed gene lists were generated using the Institutes of Genomic Research Multiexperiment Viewer (TIGR MeV, version 4.0; Saeed et al. 2003).

## Results

### DE exposure characteristics

Table 1 shows a summary of the 30-day average exposure data (4 hr/day × 5 days/week × 6 weeks) for the low (500 μg/m^3^) and high (2,000 μg/m^3^) DE concentrations. These target chamber concentrations were achieved with low variability either within a particular 4-hr exposure or between different days. CO, NO, NO_2_, and SO_2_ concentrations in the high chambers averaged 4.8, 5.9, 1.2, and 0.3 ppm, respectively. Concentrations in the low chamber were proportionally lower or below detection levels, as indicated. Geometric median number and volume (assuming spherical particles) diameters of approximately 85 and 220 nm, respectively, were measured in both chambers. It should be noted, however, that the SMPS system (with long column) limited measurements to particles greater than approximately 15 nm. OC/EC weight ratios of 0.3 from both chambers indicate that approximately 23% of the DEP was composed of organic carbon. Body weight gain for control rats was similar to those exposed to DE. Whole-body plathysmography measurement of breathing parameters revealed rat strain-dependent but no exposure-related significant differences [see Supplemental Material, Figure 1 (http://www.ehponline.org/members/2008/11647/suppl.pdf)].

### Pulmonary injury and systemic health effects of DE exposure

**Figure 1:**
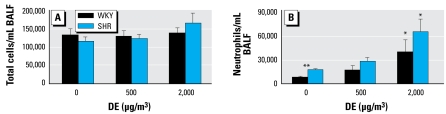
Pulmonary inflammatory response to DE as determined by analysis of BALF total cells (*A*) and neutrophils (*B*) in WKY and SH rats. Each bar represents the mean ± SE for nine animals. *Significant exposure effect.**Significant strain effect.

**Figure 2:**
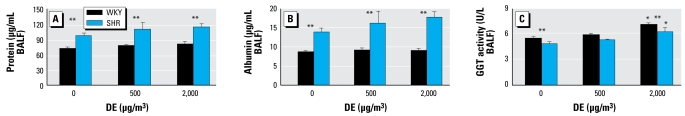
Pulmonary injury response to DE as determined by analysis of BALF levels of protein (*A*), albumin (*B*), and GGT activity in WKY and SH rats. Each bar represents the mean ± SE for nine animals. *Significant exposure effect. **Significant strain effect.

A number of pulmonary and cardiovascular biomarkers were assessed to understand their potential contribution in cardiac impact of DE [see Supplemental Material, Table 2 (http://www.ehponline.org/members/2008/11647/suppl.pdf)]. DE exposure did not result in a significant increase in total BALF cells (Figure 1A). Neutrophils increased in DE concentration-dependent manner in both strains to a similar extent (Figure 1B). The baseline neutrophil count was high in SH compared with WKY rats, as noted previously (Kodavanti et al. 2000). Strain-related differences were also apparent in BALF injury markers such as protein, albumin (SH > WKY), and GGT activity (WKY > SH) (Figure 2A–C). DE exposure did not increase BALF protein or albumin in either strain. However, a small but significant increase in GGT activity was noted in both SH and WKY rats at high DE concentration (Figure 2C). Strain differences were apparent in basal levels of several cytokines in BALF (GMCSF, IL-9, IL-18, GRO/KC, RANTES, VEGF), but no consistent exposure-related changes were apparent [see Supplemental Material, Table 3 (http://www.ehponline.org/members/2008/11647/suppl.pdf]. Similarly, no DE exposure-related changes were observed in serum cytokine (MCP-1, GMCSF, IL-9, IL-18, Gro/KC, RANTES, Leptin) levels [see Supplemental Material, Table 4 (http://www.ehponline.org/members/2008/11647/suppl.pdf)]. Plasma prothrombin time and activated partial thromboplastin time values were similar in both strains and did not change as a result of DE [see Supplemental Material, Table 5 (http://www.ehponline.org/members/2008/11647/suppl.pdf)]. As we noted earlier (Kodavanti et al. 2002), plasma fibrinogen levels were slightly higher in SH than in WKY rats. DE did not cause any change in either strain [see Supplemental Material, Table 5 (http://www.ehponline.org/members/2008/11647/suppl.pdf)]. Further, no consistent DE effects were noted in hematologic parameters. Rat strain differences were seen in hemoglobin and platelets [see Supplemental Material, Table 6 (http://www.ehponline.org/members/2008/11647/suppl.pdf)]. Also, plasma total antioxidant capacity, ACE activity, CRP, and D-dimer levels remained unaffected by DE [see Supplemental Material, Table 6 (http://www.ehponline.org/members/2008/11647/suppl.pdf)].

### Pulmonary and cardiac histology

**Figure 3:**
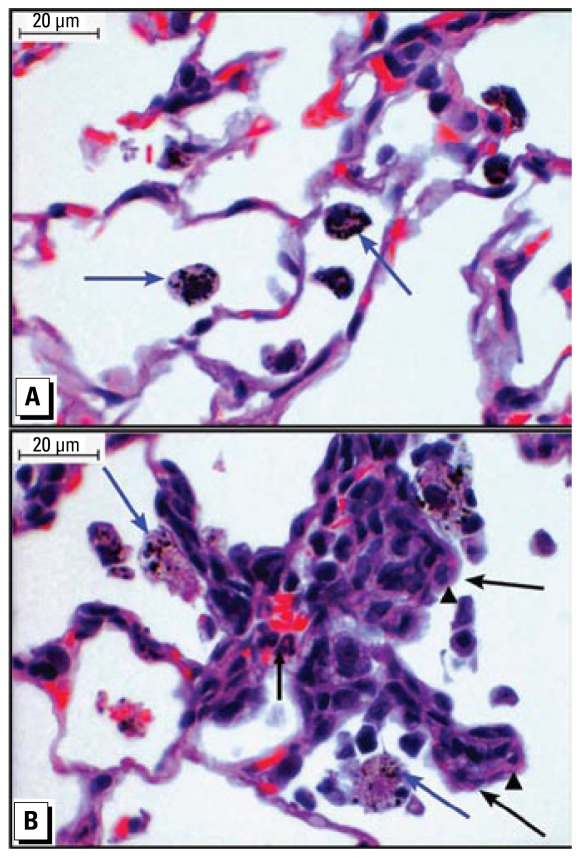
Light microscopic images of H&E-stained lung tissue sections from DE-exposed (2,000 μg/m^3^) SH rats (× 600) showing (*A*) accumulation of particle-laden macrophages (black arrows) but without extensive tissue damage, and (*B*) focal alveolar hyperplasia (black arrowhead) and inflammation (black arrows).

The macrophages contained numerous round dark DE granules of various sizes (Figure 3A; arrows) in all exposed rats. Interstitial subacute inflammation was characterized by alveolar lining epithelium cuboidal hyperplasia (Figure 3B; arrowhead) associated with interstitial mixed inflammatory cells (arrows). These foci were distributed at random but were mostly associated with the presence of alveolar histiocytosis seen in DE-exposed WKY and SH rats. Histologic evaluation of 9-step sections from each heart tissue revealed presence of focal inflammation of the pericardium or endocardium at the base in both strains regardless of DE (data not shown).

### TEM of the myocardium

**Figure 4:**
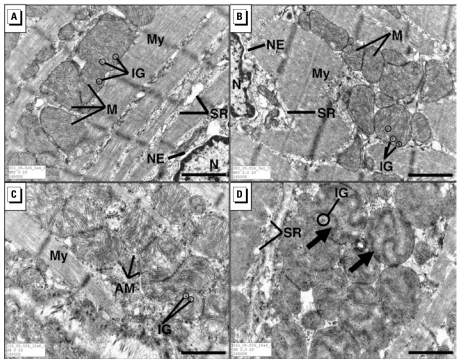
TEM findings in all three cardiac regions of WKY (*A, B*) and SH rats (*C, D*). Abbreviations: AM, abnormal mitochondria; IG, intramitochondrial granules; M, mitochondria; My, myofiber; N, nucleus; NE, nuclear envelope; SR, sarcoplasmic reticulum. (*A*) Normal appearance of cardiomyocytes and mitochondria in tissue from air-exposed WKY rats. (*B*) No significant impact of DE in WKY rats exposed to 2,000 μg/m^3^ DE. (*C*) In air-exposed SH rats, mitochondria occurred sporadically, exhibiting some electron lucency and irregularly arrayed cristae; intramitochondrial granules can be seen within mitochondria, and electron-dense ribosomes surround mitochondria. (*D*) In SH rats exposed to 2,000 μg/m^3^ DE show the disorderly arrangement of cristae within the mitochondria, which is also apparent in air-exposed rats. No DE exposure effects were readily apparent. Bar = 1 μm.

No exposure-related differences could be detected between control and DE rats within the WKY and the SH strains. Mitochondria in all groups contained electron-dense, 20- to 50-nm intramitochondrial granules, also termed dense granules, typical of these rats. In the WKY hearts, the ultrastructure of individual mitochondria exhibited good definition of all bounding inner and outer membranes and no swollen, displaced, or broken cristae (Figure 4A,B). In all three regions of both control and DE-exposed SH hearts, however, occasional mitochondrial abnormalities occurred. These were manifested as swollen, minimally to extremely electron lucent mitochondria, containing damaged or missing cristae, interspersed infrequently but conspicuously among linear arrays of normal-appearing mitochondria (Figure 4C) and mitochondria with normal-to-reduced electron density containing cristae that were sometimes arrayed irregularly, in whorls or honeycombed patterns (Figure 4D).

### Activities of cardiac cytosolic and mitochondrial enzymes

**Figure 5:**
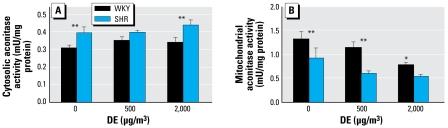
Cardiac cytosolic (*A*) and mitochondrial (*B*) aconitase activities in WKY and SH rats after exposure to DE. Each bar represents mean ± SE for six animals. *Significant exposure effect. **Significant strain effect.

There were clear strain differences (SH < WKY) in the level of glutathione peroxidase, glutathione transferase, and superoxide dismutase activities in the whole left ventricular homogenates between WKY and SH rats [see Supplemental Material, Figure 2 (http://www.ehponline.org/members/2008/11647/suppl.pdf)]. However, no DE-related differences were noted in either strain. Ferritin, an iron-binding protein, was higher in both cytosol and mitochondria of SH than WKY rats; however, no consistent DE-related changes were observed [see Supplemental Material), Figure 3 (http://www.ehponline.org/members/2008/11647/suppl.pdf]. Baseline (air control) levels of cardiac cytosolic aconitase were higher and mitochondrial aconitase activities were lower in SH than in WKY rats. DE exposure caused a dose-dependent inhibition of mitochondrial aconitase activity in both strains but did not affect the cytosolic form (Figure 5).

### DE-induced myocardial gene expression changes

We used Affymetrix rat 230A gene arrays to generate mRNA expression profiles of the left ventricular tissues for SH and WKY rats exposed to air or 2,000 μg/m^3^ DE. We found 691 genes that were significantly different (5% FDR, *p* < 0.01) between WKY-air and SH-air. DE exposure caused 377 genes to be differentially expressed within WKY rats, but surprisingly, no genes were significantly different (5% FDR, *p* < 0.01) between SH-air and SH-DE.

**Figure 6:**
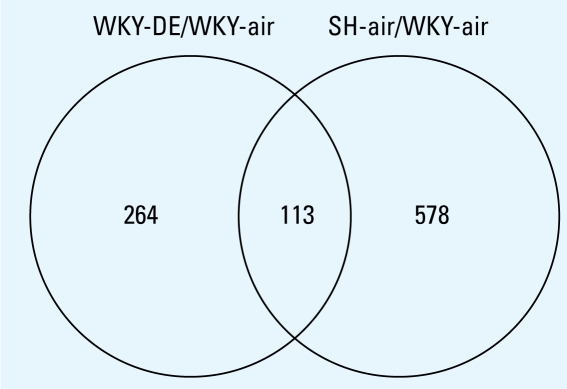
Venn diagram indicating common and differentially expressed genes in control versus DE-exposed (2,000 μg/m^3^) WKY and SH rats. Because no genes were changed by DE exposure in SH rats (5% FDR, *p* ± 0.01), only the remaining two comparisons are provided (WKY-DE/WKY-air and SH-air/WKY-air).

**Figure 7:**
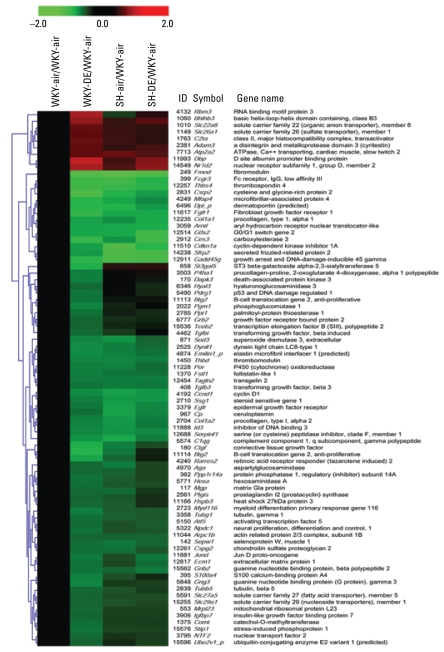
Hierarchical clustering of selected genes that are differentially expressed after DE exposure (2,000 μg/m^3^) in WKY rats. Mean values (*n* = four per group) were selected for comparing four groups. Red and green intensities indicate fold increases and decreases, respectively, in gene expression (expressed as log_2_). WKY-air group is compared with all other groups (WKY-DE, SH-air, and SH-DE). The gene ID refers to Unigene ID (Pontius et al. 2003).

Because no genes were significantly affected by DE in SH rats, only two comparisons were used (WKY-air/WKY-DE and SH-air/SH-DE) to prepare a Venn diagram for identification of common and differentially affected genes (Figure 6). Of 377 genes affected by DE in WKY rats, 113 genes were among those 691 found different between SH and WKY rats at baseline without DE (Figure 6). Two hundred sixty-four genes affected by DE in WKY were among those found to be insignificant with regard to strain difference at baseline. We used the PubMed database to perform a functional scrutiny of the genes that were up-regulated ≥ 1.4-fold and down-regulated ≤ 1.4-fold within the group of 377 significant genes, which allowed us to focus on more robust effects and select 80 genes changed by DE exposure in WKY rats for hierarchical cluster analysis. The heat map (Figure 7), although focused on 80 genes affected by DE in WKY rats, included all four contrasts (WKY-air/WKY-air; WKY-DE/WKY-air; SH-air/WKY-air; SH-DE/WKY-air). This heat map allowed us to determine how these genes are related to the baseline expression pattern in air-exposed SH rats (Figure 7).

**Figure 8:**
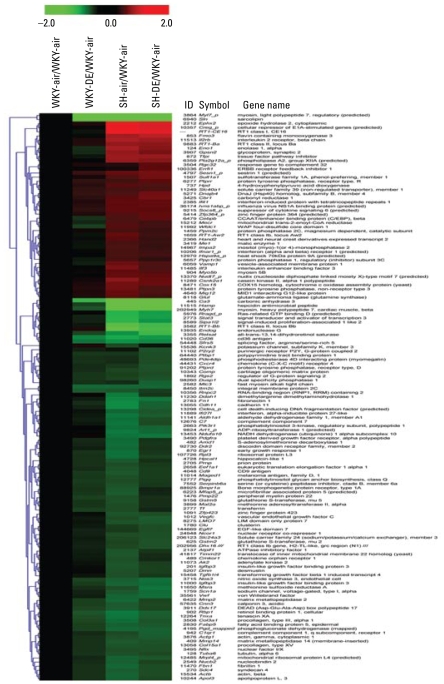
A heat map showing strain-related differences in expression pattern as fold-change intensities from group contrasts with WKY-air control. Red and green intensities indicate fold increases and decreases, respectively, in gene expression (expressed as log_2_). For this heat map, significant genes for SH-air/WKY-air were selected, and those included in the first heat map were deleted. The WKY-air group was compared with all other groups (air and 2,000 μg/m^3^ DE in both strains). The gene ID refers to unigene ID (Pontius et al. 2003).

We prepared a second heat map (Figure 8) from genes that were differentially expressed in SH-air relative to the WKY-air but devoid of the selected 80 genes included in the previous heat map. This list included genes that were up-regulated ≥ 1.5-fold or down-regulated ≤ 1.5-fold (5% FDR, *p* < 0.01) and further scrutinized for functional significance based on published literature, which resulted in 137 genes for hierarchical cluster analysis (Figure 8). This heat map (Figure 8) also examined the same contrasts as in the first heat map (Figure 7).

When we compared genes that were differentially expressed between WKY-air and SH-air rats (Figures 7 and 8), significant strain differences were noticeable, as expected, based on underlying cardiac disease in SH rats versus a healthy pattern in WKY rats. The most striking observation, however, was that DE exposure in WKY rats resulted in an expression pattern shift, especially for 113 genes of 377 affected by DE, that mimicked baseline expression pattern in SH rats without DE exposure. Examination of the pattern change in the heat map (Figure 7) showed that the WKY-DE group was more closely related to the SH-air group than the WKY-air group. Evaluation of the functional roles of these changed genes in WKY rats indicated their involvement in hypertensive and cardiovascular disorder in humans and animal models. Further, most genes affected by DE in WKY rats were down-regulated and very few were up-regulated (Figure 7). These genes were primarily among 113 genes that were also down-regulated at baseline in SH rats without DE (Figure 7). They include those involved in stress, antioxidant compensatory response, growth and extracellular matrix regulation, membrane transport of molecules including lipids, mitochondrial function, thrombosis regulation, and immune function (Figure 7) [see Supplemental Material, Table 7 (http://www.ehponline.org/members/2008/11647/suppl.pdf)]. Genes that are crucial in vascular tone (NO synthase) and pathology (sarcolipin, apolipoprotein L, 3) were also slightly down-regulated by DE in WKY and at baseline in air-exposed SH rats (Figure 8). Those up-regulated by DE include RNA-binding motif protein 3, ion transporters, and calcium transporting ATPase. Except for RNA-binding motif protein 3 gene, SH also demonstrated an upward trend at baseline with no DE effect in relation to air-exposed WKY.

Genes demonstrating increased expression at baseline in SH rats relative to WKY-air are not those that show significant increase with DE in WKY rats except for a notable upward trend in epoxide hydrolase 2, interleukin-2 receptor, sastrin-1 predicted protein tyrosine phosphatase, and a few other genes (Figure 8). Evaluation of the functional role of these genes may explain the baseline cardiac pathology (Figure 4) and biochemical alterations in SH but not produced by DE in WKY rats (Figure 5) [see Supplemental Material, Figures 2 and 3 (http://www.ehponline.org/members/2008/11647/suppl.pdf)]. These genes (Figure 8) include those involved in mitochondrial function, fatty acid metabolism, cytokine signaling, and stress response. Two-thirds of the selected genes in the heat map (Figure 8) were down-regulated in SH at baseline when compared with the WKY-air group. Although not necessarily significant (5% FDR, *p* ± 0.05), many of those genes also showed a downward trend in WKY rats exposed to DE.

## Discussion

DE, a major component of near-road and ambient PM, has consistently shown cardiovascular impairment in clinical and experimental studies (Campen et al. 2005; Carlsten et al. 2007; Hansen et al. 2007; Mills et al. 2005, 2007; Tornqvist et al. 2007; Urch et al. 2005). However, the mechanisms that produce cardiovascular functional impairment, such as endothelial dysfunction and hypertension, are not well understood. We provide evidence that DE inhalation produces hypertensive-like gene expression pattern associated with mitochondrial oxidative stress in ventricles of healthy rats. A robust DE effect was apparent in WKY (377 genes with 5% FDR correction and significance at *p* < 0.01) but minimal effect was evident in SH rats. More importantly, the directional changes in expression pattern after DE exposure in WKY rats appear to mimic expression pattern of SH rats at baseline (air group). Because our primary objective was to compare susceptibility of SH and WKY emphasizing the role of oxidative stress, we did not measure blood pressure in this study. However, increased blood pressure, vasoconstriction, and endothelial dysfunction have been reported in both humans and animals after exposure to DE or other PM (Brook 2005; 2007; Elder et al. 2007; Hansen et al. 2007; Knuckles et al. 2008; Peretz et al. 2008). Further, many genes that were affected by DE in WKY were down-regulated, and only a few were up-regulated. These are the same genes down-regulated in SH rats without DE, suggesting that this pattern is associated with down-regulation of a group of genes that may be considered susceptibility genes. Although a number of key genes related to mitochondrial function and compensatory response to oxidative stress were found impaired by DE, supporting the role of oxidative stress and mitochondrial aconitase inhibition, the most striking inhibition was noted in genes that regulate growth and matrix components. A mechanistic explanation for the role of structural genes cannot be ascertained from this experiment, but one may associate this inhibitory response to cardiac physiologic impairment without histologically demonstrable fibrosis or inflammatory response in the DE-exposed WKY rats. Matrix components in the heart not only maintain structural integrity but also regulate muscle contraction (Ota et al. 2007). Thus, we provide novel evidence of development of hypertensive-like gene expression pattern associated with increased oxidative stress without apparent morphologic alteration that might explain human clinical and epidemiologic findings of cardiovascular physiologic impairment (i.e., increased vasoconstriction and blood pressure) after DE exposure.

At TEM level, no DE effects could be distinguished in the cardiac mitochondria of either rat strain. However, among many oxidative stress-sensitive enzymes analyzed, dose-related inhibition of cardiac mitochondrial aconitase was apparent in both strains exposed to DE, suggesting mild oxidative stress and likely impaired energy production. Mitochondrial oxidative stress has been suggested as the mechanism of PM-induced cardiac injury (Xia et al. 2007). Lower mitochondrial aconitase activity in the hearts of SH rats relative to WKY at baseline is consistent with observed ultrastructural abnormalities in cardiac mitochondria and underlying cardiac oxidative stress in hypertensive rats (Friedman et al. 2003). Cardiac mitochondrial aconitase activity inhibition without structural deficits in DE-exposed WKY as opposed to structural deficits with aconitase inhibition in SH rats may suggest that more chronic oxidative stress and other genetic predisposition may be critical in producing these abnormalities.

Although cardiac mitochondrial oxidative stress and other pulmonary effects were dose dependent, gene expression was analyzed only for the high concentration group (2,000 μg/m^3^). The concentrations of DE used in this study were much higher than those encountered in ambient air; however, these concentrations have been used experimentally (Risom et al. 2007) and may be achieved during heavy traffic or occupational situations. In the absence of consistent data on PM causing any molecular or biochemical changes in the heart, we wanted to provide the highest possible insult to prove that cardiac pathobio-chemical and molecular effects do occur after pulmonary insult, not only in compromised but also in healthy rats. We believe that high concentration exposures allow demonstration of a clear distinction of DE effects between SH and WKY rats. Further, this concentration resulted in only mild lung inflammation and no consistent systemic effects, much like what is expected after high-level pollution exposure.

It is not clear how pulmonary inflammatory changes may secondarily induce cardiac toxicity, but one may speculate that low-level, long-term pulmonary inflammation causes microvascular impairment and changes vascular tone as well as cardiac contractility. However, we previously reported that WKY rats exposed to Mount St Helen’s ash, residual oil fly ash, or soluble zinc sulfate once weekly over 8 or 16 weeks, although having remarkable pulmonary pathology and slight increase in background levels of cardiac lesions, did not demonstrate as remarkable cardiac gene expression changes (Kodavanti et al. 2008) as seen presently in DE-exposed WKY rats. Most changes in gene expression in the previous study were dissimilar to the present study and appeared zinc specific, as they occurred primarily in rats exposed to zinc sulfate. Our recent subchronic zinc inhalation study at environmentally and occupationally relevant zinc levels further confirmed zinc-specific effects on cardiac gene expression that were dissimilar to what we have seen in DE-exposed WKY rats with regard to the types of genes affected; they were also of smaller magnitude (Wallenborn et al., in press) than those observed in the present study. Thus, it appears that DE effects on cardiac gene expression are unique to freshly generated whole DE, which includes gaseous components, and not related to the extent of lung pathology or mere presence of particles in the lung. This is not to undermine the fact that chronic pulmonary pathology can secondarily affect cardiac structure and function, but with the present experimental design, the effects seen in the heart appear DE specific.

It has been also shown that reactive polycyclic hydrocarbons adherent to DE can rapidly translocate to circulation and cause direct vascular effects and subsequent cardiac alterations (Persson et al. 2002). DE exposure has been shown to reduce vascular NO production and promote vasoconstriction (Campen et al. 2005; Knuckles et al., 2008). Our study demonstrated slightly reduced expression of endothelial NOS in the hearts of WKY rats, which may suggest that DE alters systemic vascular contraction by altering NO production in vessels and heart. It remains to be confirmed if endothelial NOS mRNA reduction in the ventricles of DE-exposed WKY rats reflects the effect on vessels within heart tissue. Because endothelial NOS expression was also down-regulated in already hypertensive SH rats at baseline, it is likely that DE exposure might cause vasoconstriction in WKY rats via its vascular effects. However, this hypothesis needs to be confirmed with protein and physiologic measurements. As our primary hypothesis was to investigate the role of mitochondrial oxidative stress and determine how gene expression patterns differ between two rat strains after DE exposure, and also because our findings of DE effects on gene expression are unexpected, we did not determine blood pressure in the present study. Future studies should determine cardiac physiologic impact of DE exposure along with gene expression using acute and chronic exposure scenarios.

In the present study we could not distinguish the effects of gaseous versus particulate DE fraction in causing cardiac effects, as our exposure to freshly generated whole DE was with concentration-dependent differences in the levels of gaseous components such as carbon monoxide, sulfur dioxide, and nitrogen oxides. DE gaseous and particulate components are released in the air, and exposure to whole DE is the most realistic scenario. However, this will not allow us to identify causative components or the role of atmospheric aging of DE. Our previous studies evaluated the role of PM-associated leachable metals in cardiac injury, more specifically zinc (Kodavanti et al. 2008; Wallenborn et al. 2008). However, DE generated using a stationary engine and compressor is less likely to have significant quantities of leachable metals or zinc (Wichmann 2007) that can cause acute cardiac injury at concentrations likely achieved in the present study. We did not measure leached-off elemental components in the present study. Most DE effects have been attributed to organic components and carbon (Singh et al. 2004; Wichmann 2007). The compositions of DE are likely to vary depending on the engine design, fuel composition, and elemental and organic carbon contents (Singh et al. 2004). Thus, the extent of response seen our study may vary depending on the DE sample tested.

A number of our studies have attempted to determine cardiac pathology, hematology, and systemic markers of cardiac injury after acute or subchronic pulmonary PM exposure (Kodavanti et al. 2003, 2008; Wallenborn et al. 2007). However, in most cases, mild to negative findings are obtained. This could be due to the insensitivity of these markers to subtle cardiac injury caused by PM under variable background of the host species. It is also likely that 1-month DE exposure might just alter cell response, leading to change in expression pattern, and may not produce classical signs of cardiac inflammation, hypertrophy, or fibrosis that can be discerned by histologic analysis of cardiac tissues. To determine the extent of cardiovascular injury caused by PM, in general, classical markers of inflammation up-regulated in most disease conditions are analyzed. DE exposure did not affect genes involved in inflammatory response in the heart, but led to down-regulation of numerous genes critical in compensatory response. Down-regulation of compensatory mechanisms and its association to mitochondrial impairment needs to be further investigated using acute and chronic exposure scenarios.

Although no expression changes were apparent in SH rats after DE exposure, determining expression profiles for these rats in parallel to WKY provided novel insights into the potential mechanism of cardiac DE effects. One likely explanation for the lack of demonstrable effect in SH rats is that the genes down-regulated by DE were already repressed in control SH rats and therefore a further DE effect was not demonstrable. To understand the significance of this down-regulation, it is important to study these genes in the context of cardiovascular disease. Genes that were down-regulated include those involved in stress response, compensatory mechanisms, growth factors, and extra cellular matrix components. Notable examples of inhibited matrix metabolism genes include cycline kinase inhibitors, TGF-β, and several downstream matrix components. Cycline kinase inhibitors regulate matrix production in vascular smooth muscles cells via TGF signaling (Weiss and Randour 2002). Thus, cycline kinase-mediated inhibition of TGF signaling may provide a potential mechanism by which matrix metabolism is altered by DE. Exposure to gasoline exhaust has been shown to up-regulate expression of matrix metalloproteinases in mouse aorta (Lund et al. 2006). We did not observe a noticeable pattern of altered expression of metalloproteinases in the hearts of DE-exposed WKY rats, which warrants examination of vascular tissues such as aorta. In hypertensive rats, Wang et al. (2004) observed a few matrix genes that were down-regulated early in the development of hypertension (similar to the age group used in the present study). Although it is not clear from our study how critical the down-regulated matrix genes are, alteration in myocardial contractility has been linked to matrix abnormalities (Ota et al. 2007). These expression changes are dissimilar to previously observed changes in the hearts of WKY rats after episodic zinc exposure (Kodavanti et al. 2008), suggesting that hypertensive pattern development may be specific to whole DE exposure and may contribute to vaso-constriction in near-road residents.

The major question that remains is why DE exposure did not change expression of any genes in SH rats. This may imply that already compensated hearts of SH may not be able to respond to DE in a manner similar to hearts of WKY, despite their greater sensitivity to plasma fibrinogen increase and pulmonary injury/oxidative stress (Kodavanti et al. 2002, 2005). The lack of demonstrable down-regulation of already down-regulated genes supports the idea that a larger insult may be needed for SH rats to be able to respond to DE. The other possible explanation for reduced sensitivity of SH rats, despite pulmonary neutrophilic inflammation being similar or greater than WKY, may include strain differences in the retention and clearance of DE from lung and the importance of longer particle presence in the lung to elicit a systemic response. To understand these mechanisms, further studies are needed to evaluate DE-clearance kinetics and the contribution of the affected genes at protein level in parallel to blood pressure measurement after DE exposure in SH and WKY rats.

## Conclusion

Our study provides novel insights into DE-induced cardiac effects and the susceptibility of the compromised heart. We report that 1-month DE exposure enhances cardiac mitochondrial oxidative stress in both healthy and hypertensive rats. This oxidative stress is associated with development of a hypertensive-like cardiac gene expression pattern in healthy rats while hearts of already hypertensive rats are spared from DE-induced further impairment in gene expression. DE exposure primarily caused down-regulation of genes already down-regulated in control SH rats at baseline but largely spared those already increased in SH rats at baseline. Thus, we provide novel evidence of potential mechanisms by which DE may produce cardiac effects in healthy and cardiovascular-compromised rats that explain physiologic impairments observed in humans.

## Figures and Tables

**Table 1:** Summary of concentrations and characteristics of the DE particles and gases within the animal exposure chambers.^a^

Constituent (unit)	Low exposure	High exposure
Particle mass concentration by TEOM (μg/m^3^)	507 ± 4	2,201 ± 14
Particle mass concentration (filter)^b^ (μg/m^3^)	467 ± 16	2,037 ± 23
Particle number concentration^c^ (no./cm^3^)	6.1 × 10^5^ ± 8.8 × 10^4^	1.5 × 10^6^ ± 1.6 × 10^5^
O_2_ (%)	20.6 ± 0.02	20.4 ± 0.02
CO (ppm)	1.3 ± 0.07	4.8 ± 0.26
NO (ppm)	< 2.5	5.9 ± 0.28
NO_2_ (ppm)	< 0.25	1.2 ± 0.31
SO_2_ (ppm)	0.2 ± 0.01	0.3 ± 0.01
Number median D_p_^d^ (nm)	83 ± 2	88 ± 2
Volume median D_p_ (nm)	207 ± 2	225 ± 2
OC/EC^e^ (wt ratio)	0.3 ± 0.03	0.3 ± 0.03
Temperature (°C)	22.4 ± 0.06	22.2 ± 0.06
Relative humidity (%)	54.8 ± 0.30	60.8 ± 0.30

TEOM, tapered element oscillating microbalance.

a O_2_, CO, NO, NO_2_, SO_2_, temperature, and relative humidity data represent mean ± SE from continuous measurements taken over the entire 30-day (4 hr/day × 5 days/week × 6-week) exposure.

b Filter data represent mean ± SE from one measurement per day taken over the 6-week exposure.

c Particle number concentration data represent mean ± SE from one representative measurement per week for both low and high exposure chambers taken over the 6-week exposure.

d D_p_ indicates particle geometric number and volume median diameters for a single representative particle size distribution ± geometric standard deviation. Volume information is calculated from number-based mobility diameters and assume spherical particles.

e OC/EC data represent mean ± SE from one measurement per day taken over the 6-week exposure.
